# Thrombin induces heme oxygenase-1 expression in human synovial fibroblasts through protease-activated receptor signaling pathways

**DOI:** 10.1186/ar3815

**Published:** 2012-04-27

**Authors:** Ju-Fang Liu, Sheng-Mou Hou, Chun-Hao Tsai, Chun-Yin Huang, Wei-Hung Yang, Chih-Hsin Tang

**Affiliations:** 1 Central Laboratory, Shin-Kong Wu Ho-Su Memorial Hospital, 95 Wen Chang Road, Taipei, Taiwan; 2 Department of Orthopedic Surgery, Shin-Kong Wo Ho-Su Memorial Hospital, 95 Wen Chang Road, Taipei, Taiwan; 3 Department of Orthopaedic Surgery, China Medical University Hospital, 91 Hsueh-Shih Road, Taichung, Taiwan; 4 School of Medicine and Graduate Institute of Clinical Medical Science, China Medical University, 91 Hsueh-Shih Road, Taichung, Taiwan; 5 Department of Orthopaedic Surgery, China Medical University Beigang Hospital, 123 Hsin Te Road, Yun-Lin County, Taiwan; 6 Department of Orthopedic Surgery, Taichung Hospital, Department of Health, 1 San Min Road, Taichung, Taiwan; 7 Graduate Institute of Biotechnology, National Chung Hsing University, 250 Kuo Kuang Road, Taichung, Taiwan; 8 School of Chinese Medicine, China Medical University, 91 Hsueh-Shih Road, Taichung, Taiwan; 9 Department of Pharmacology, School of Medicine, China Medical University, 91 Hsueh-Shih Road, Taichung, Taiwan; 10 Graduate Institute of Basic Medical Science, China Medical University, 91 Hsueh-Shih Road, Taichung, Taiwan

## Abstract

**Introduction:**

Thrombin is a key factor in the stimulation of fibrin deposition, angiogenesis, and proinflammatory processes. Abnormalities in these processes are primary features of osteoarthritis (OA). Heme oxygenase (HO)-1 is a stress-inducible rate-limiting enzyme in heme degradation that confers cytoprotection against oxidative injury. Here, we investigated the intracellular signaling pathways involved in thrombin-induced HO-1 expression in human synovial fibroblasts (SFs).

**Methods:**

Thrombin-mediated HO-1 expression was assessed with quantitative real-time (q)PCR. The mechanisms of action of thrombin in different signaling pathways were studied by using Western blotting. Knockdown of protease-activated receptor (PAR) proteins was achieved by transfection with siRNA. Chromatin immunoprecipitation assays were used to study *in vivo *binding of Nrf2 to the HO-1 promoter. Transient transfection was used to examine HO-1 activity.

**Results:**

Osteoarthritis synovial fibroblasts (OASFs) showed significant expression of thrombin, and expression was higher than in normal SFs. OASFs stimulation with thrombin induced concentration- and time-dependent increases in HO-1 expression. Pharmacologic inhibitors or activators and genetic inhibition by siRNA of protease-activated receptors (PARs) revealed that the PAR1 and PAR3 receptors, but not the PAR4 receptor, are involved in thrombin-mediated upregulation of HO-1. Thrombin-mediated HO-1 expression was attenuated by thrombin inhibitor (PPACK), PKCδ inhibitor (rottlerin), or c-Src inhibitor (PP2). Stimulation of cells with thrombin increased PKCδ, c-Src, and Nrf2 activation.

**Conclusion:**

Our results suggest that the interaction between thrombin and PAR1/PAR3 increases HO-1 expression in human synovial fibroblasts through the PKCδ, c-Src, and Nrf2 signaling pathways.

## Introduction

Osteoarthritis (OA) is a degenerative disease characterized by a slow progressive degeneration of articular cartilage, variable secondary synovial inflammation, and osteophyte formation [1,2]. The exact etiology of OA is not well understood. In response to macrophage-derived proinflammatory cytokines, such as interleukin (IL)-1β and tumor necrosis factor-α (TNF)-α, OA synovial fibroblasts (OASFs) produce chemokines that promote inflammation, neovascularization, and cartilage degradation through activation of matrix-degrading enzymes such as matrix metalloproteinases (MMPs) [3].

Heme oxygenase (HO)-1 is the key enzyme responsible for the degradation of heme to carbon monoxide, free iron, and biliverdin-IX [4]. In mammals, biliverdin-IX is further converted to bilirubin-IX, an endogenous radical scavenger with recently recognized antiinflammatory properties [5]. Free iron is rapidly sequestered into the iron-storage protein ferritin, leading to additional antioxidant and antiapoptotic effects [6]. Carbon monoxide serves several biologic functions, with antiapoptotic and antiinflammatory properties [7]. HO-1 is induced by various stimuli, such as lipopolysaccharide (LPS), proinflammatory cytokines, and oxidants [8,9]. Recent findings revealed that HO-1 induction in animals protects them against the development of arthritis [10]. HO-1 induction in OA chondrocytes results in decreased levels of MMPs and can exert protective effects against cartilage degradation [11]. In synovial cells, HO-1 is an important factor regulating inflammation and cartilage degradation during OA [12]. Therefore, HO-1 plays a crucial role in OA pathogenesis. However, the role of thrombin in HO-1 expression in OA is still unknown.

Thrombin is a multifunctional enzyme that can activate hemostasis and coagulation through the cleavage of fibrinogen to form fibrin clots. Tissue damage and fibrin deposition are common features of inflamed synovium in OA, which indicates generation of thrombin in the lesions and suggests an involvement of thrombin in the joint inflammation [13]. Thrombin also acts as a mitogen to stimulate the abnormal proliferation of synovial cells during rheumatoid arthritis (RA) and OA pathogenesis [14,15]. Thrombin activates intracellular signaling pathways by interacting with transmembrane domain G protein-coupled receptors, known as protease-activated receptors (PARs). PARs have been implicated in the development of acute and chronic inflammatory responses. Three PARs, PAR-1, PAR-3, and PAR-4, are cleaved by thrombin, whereas PAR-2 is cleaved by trypsin. It has been reported that thrombin induces HO-1 release in human microglia [16], indicating that thrombin may play a role in the regulation of specific gene expression, such as that of HO-1. However, the effect of thrombin on HO-1 expression in human synovial fibroblasts has not yet been elucidated. In the present study, we explored the intracellular signaling pathways involved in thrombin-induced HO-1 expression in human OASFs. In our experiment, thrombin activated the PAR1/PAR3 receptor, PKCδ, c-Src, and Nrf2 pathways, leading to the upregulation of HO-1 expression. These results provide new insights into the mechanisms of thrombin action that may be of therapeutic value for OA.

## Materials and methods

### Materials

Protein A/G beads, anti-mouse and anti-rabbit IgG-conjugated horseradish peroxidase, rabbit polyclonal antibodies specific for thrombin, PAR1, PAR3, PAR4, c-Src, PKCδ, and Nrf2, and siRNA against PAR1, PAR3, and PAR4 were purchased from Santa Cruz Biotechnology (Santa Cruz, CA, USA). Rabbit polyclonal antibody specific for c-Src phosphorylated at Tyr^416 ^and PKCδ phosphorylated at Tyr^331 ^were purchased from Cell Signaling and Neuroscience (Danvers, MA, USA). Rabbit polyclonal antibody specific for Nrf2 phosphorylated at Ser^40 ^was purchased from Abcam Inc. (Cambridge, MA, USA). SFLLRN-NH_2 _(a PAR1-agonist peptide), TFRGAP-NH_2 _(a PAR3-agonist peptide), and GYPGQV-NH_2 _(a PAR4-agonist peptide) were purchased from Bachem. Rottlerin, GF109203X, Ro320432, and PP2 were purchased from Calbiochem (Darmstadt, Germany). The c-Src dominant-negative mutant was a gift from Dr. S. Parsons (University of Virginia Health System, Charlottesville, VA, USA). The human HO-1 promoter construct was gift from Dr. Y.C. Liang (Taipei Medical University, Taipei, Taiwan). The pSV- β-galactosidase vector and luciferase assay kit were purchased from Promega (Madison, WI, USA). All other chemicals were purchased from Sigma-Aldrich (St. Louis, MO, USA).

### Cell cultures

We obtained approval from the local ethics committee, and subjects gave informed written consent. Human synovial fibroblasts were isolated by collagenase treatment of synovial tissue samples obtained from 32 patients with OA during knee-replacement surgeries and 18 samples of nonarthritic synovial tissues obtained at arthroscopy after trauma/joint derangement. The concentration of thrombin in the synovial fluid of selected patients was measured with an enzyme-linked immunosorbent assay (ELISA) according to the protocol provided by the manufacturer (Human thrombin ELISA kit; Abcam). OASFs were isolated, cultured, and characterized, as previously described [17,18]. Experiments were performed by using cells from passages 3 to 6.

### Quantitative real-time PCR

Total RNA was extracted from synovial fibroblasts by using a TRIzol kit (MDBio Inc., Taipei, Taiwan). The reverse-transcription reaction was performed by using 2 μg of total RNA that was reverse transcribed into cDNA by using oligo (dT) primer [19,20]. The quantitative real-time PCR (qPCR) analysis was carried out by using Taqman one-step PCR Master Mix (Applied Biosystems, Foster City, CA, USA). cDNA templates (2 μl) were added per 25-μl reaction with sequence-specific primers and Taqman probes. Sequences for all target gene primers and probes were purchased commercially (β-actin was used as internal control) (Applied Biosystems). The qPCR assays were carried out in triplicate on a StepOnePlus sequence-detection system. The cycling conditions involved 10-minute polymerase activation at 95°C, followed by 40 cycles at 95°C for 15 seconds and 60°C for 60 seconds. The threshold was set above the nontemplate control background and within the linear phase of the target gene amplification to calculate the cycle number at which the transcript was detected (denoted CT).

### Western blot analysis

Cellular lysates were prepared as described previously [21,22]. Proteins were resolved on SDS-PAGE and transferred to Immobilon polyvinyldifluoride (PVDF) membranes. The blots were blocked with 4% BSA for 1 hour at room temperature and then probed with rabbit anti-human antibodies against PKCδ, c-Src, or Nrf2 (1:1,000) for 1 hour at room temperature. After three washes, the blots were subsequently incubated with donkey anti-rabbit peroxidase-conjugated secondary antibody (1:3,000) for 1 hour at room temperature. The blots were visualized by enhanced chemiluminescence with Kodak X-OMAT LS film (Eastman Kodak, Rochester, NY, USA).

### Transfection and reporter gene assay

Human synovial fibroblasts were co-transfected with 0.8 μg HO-1-luciferase plasmid and 0.4 μg β-galactosidase expression vector. Fibroblasts were grown to 80% confluence in 12-well plates and were transfected the following day with Lipofectamine 2000 (LF2000; Invitrogen). DNA and LF2000 were premixed for 20 minutes and then applied to cells. After 24-hour transfection, cells were incubated with the indicated agents. After further 24-hour incubation, the media were removed, and cells were washed once with cold PBS. To prepare lysates, 100 μl of reporter lysis buffer (Promega, Madison, WI, USA) was added to each well, and cells were scraped from dishes. The supernatant was collected after centrifugation at 13,000 rpm for 2 minutes. Aliquots of cell lysates (20 μl) containing equal amounts of protein (20 to 30 μg) were placed into wells of an opaque black 96-well microplate. An equal volume of luciferase substrate was added to all samples, and luminescence was measured in a microplate luminometer. The value of luciferase activity was normalized to transfection efficiency monitored by the co-transfected β-galactosidase expression vector [23].

### Kinase-activity assay

PKCδ activity was assessed with a PKCδ Kinase Activity Assay Kit according to manufacturer's instructions (Assay Designs, MI, USA). The PKCδ activity kit is based on a solid-phase ELISA that uses a specific synthetic peptide as a substrate for PKCδ and a polyclonal antibody that recognizes the phosphorylated form of the substrate.

### Chromatin immunoprecipitation assay

Chromatin immunoprecipitation (ChIP) analysis was performed as described previously [24]. DNA immunoprecipitated by anti-Nrf2 antibody was purified. The DNA was then extracted with phenol-chloroform. The purified DNA pellet was subjected to PCR. PCR products were then resolved by using 1.5% agarose gel electrophoresis and visualized with UV. The primers: 5'-CCATCAAACTTTAACTCGGTGA-3' and 5'- GACTTGGGAGATAGAAGGAACG-3' were used to amplify across the human HO-1 promoter region (-857 to -752) [24].

### Statistics

The values are reported as mean ± SEM. Statistical analysis between two samples was performed by using the Student *t *test. Statistical comparisons of more than two groups were performed by using one-way analysis of variance (ANOVA) with the Bonferroni *post hoc *test. In all cases, *P *< 0.05 was considered significant.

## Results

### Thrombin induces HO-1 expression in human synovial fibroblasts

**Figure 1:**
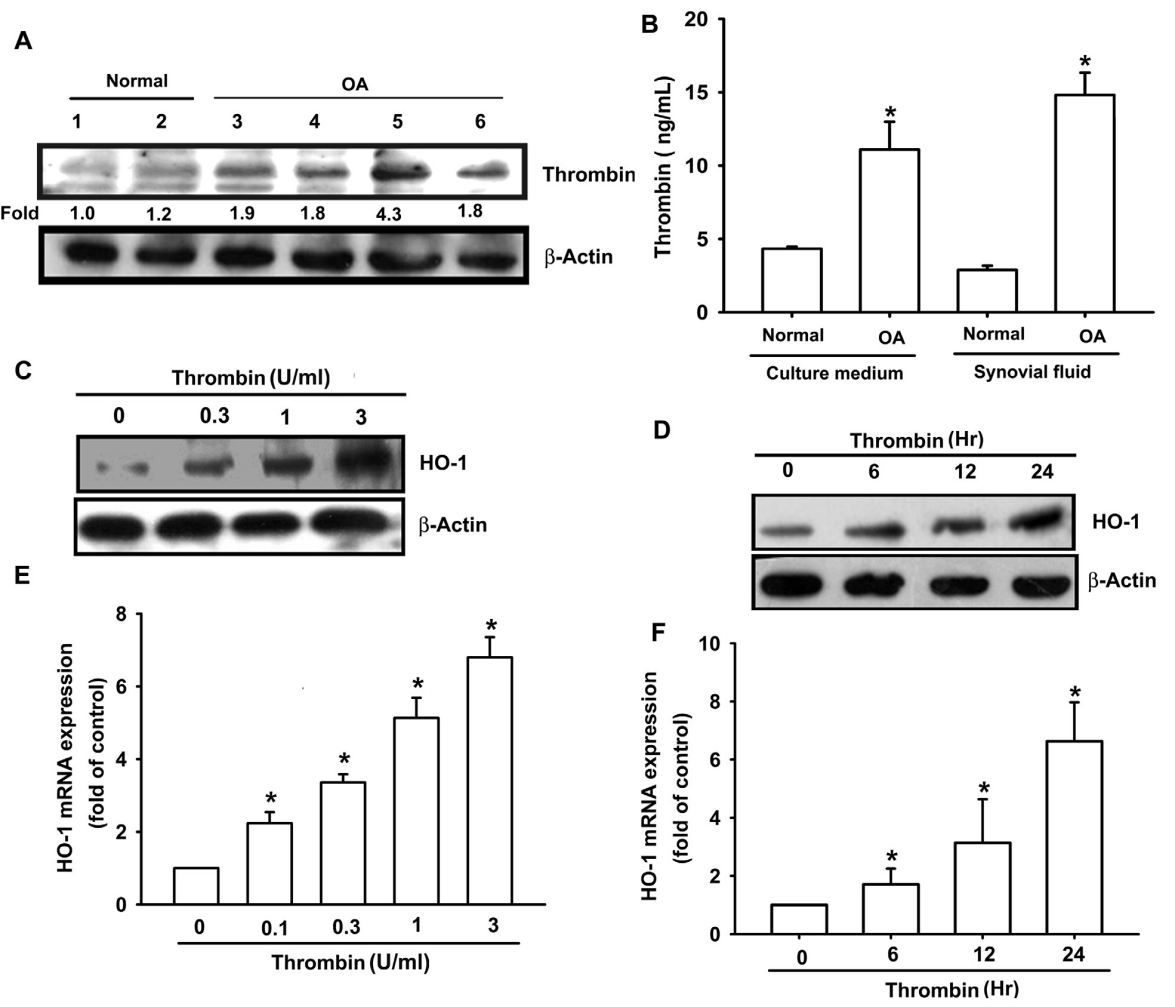
**Thrombin induces HO-1 expression in human synovial fibroblasts**. **(A) **Total protein was extracted from normal synovial fibroblasts (lines 1 and 2) and osteoarthritis synovial fibroblast (OASF) cells (lines 3 to 6), and subjected to Western blot analysis for thrombin. **(B; left panel) **Human synovial fibroblasts were cultured for 48 hours, and media were collected to measure thrombin. **(B; right panel) **Synovial fluid was obtained from normal (*n *= 10) or osteoarthritis patients (*n *= 15) and examined with ELISA for the expression of thrombin. OASFs (six OA lines) were incubated with various concentrations of thrombin for 24 hours **(C, E) **or with thrombin (3 U/ml) for 6, 12, or 24 hours **(D, F)**. The HO-1 expression was examined with Western blotting and qPCR. Results are expressed as the mean ± SEM. *P < 0.05 as compared with basal level. #*P *< 0.05 as compared with the thrombin-treated group.

**Figure 2:**
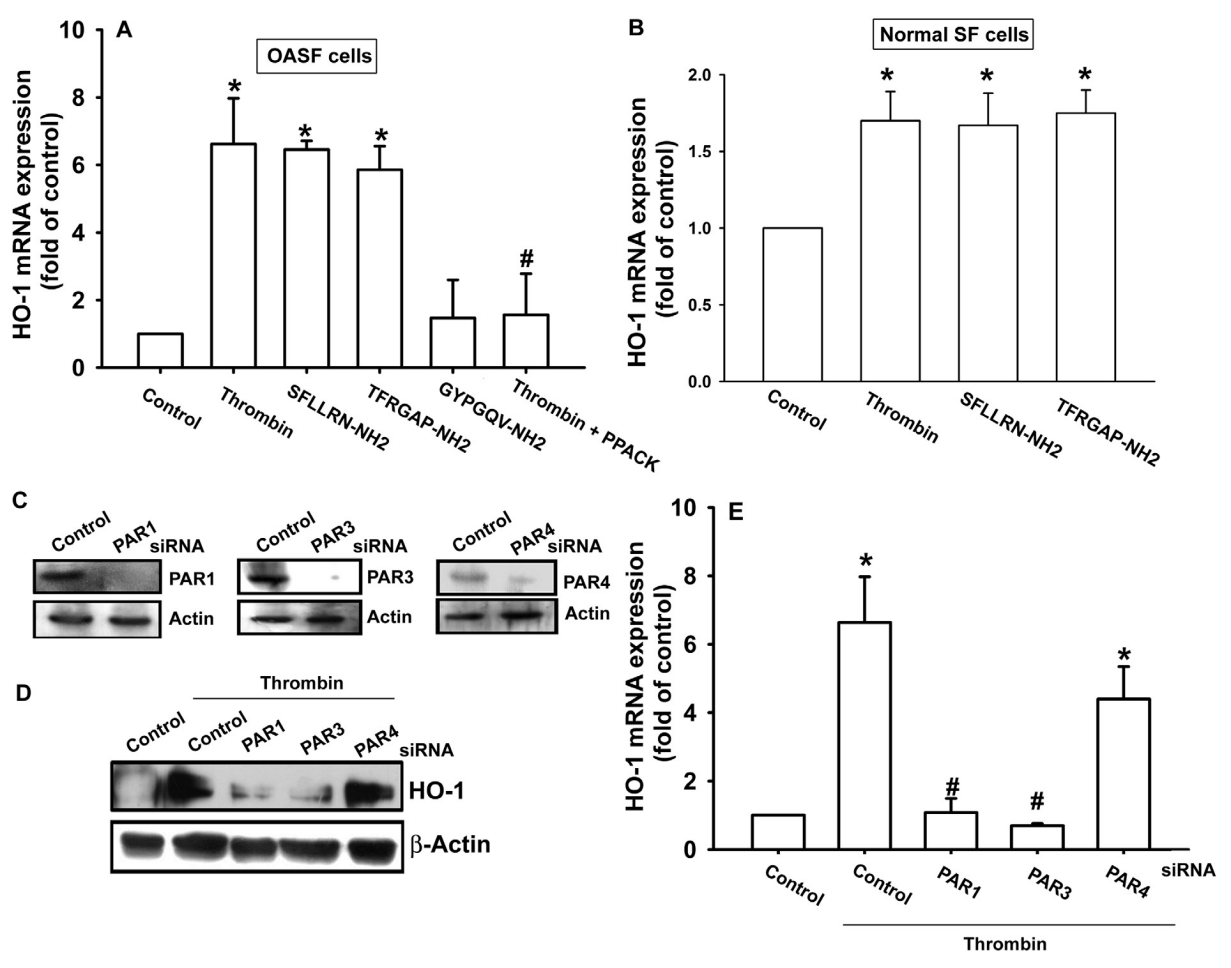
**Involvement of PAR1 and PAR3 receptor in thrombin-induced HO-1 expression**. **(A) **Osteoarthritis synovial fibroblasts (OASFs) (six OA lines) were treated with thrombin (3 U/ml), thrombin plus PPACK (30 n*M*), SFLLRN-NH_2 _(100 μ*M*), TFRGAP-NH_2 _(100 μ*M*), and GYPGQV-NH_2 _(100 μ*M*) for 24 hours, and HO-1 expression was examined with qPCR. **(B) **Normal synovial fibroblasts (seven normal lines) were treated with thrombin (3 U/ml), SFLLRN-NH_2 _(100 μ*M*), and TFRGAP-NH_2 _(100 μ*M*) for 24 hours, and HO-1 expression was examined with qPCR. **(C) **Cells were transfected with PAR1, PAR3, PAR4, or control siRNA for 24 hours, and PAR receptor level was examined with Western blotting. **(D, E) **Cells (five OA lines) were transfected with PAR1, PAR3, PAR4, or control siRNA for 24 hours followed by stimulation with thrombin for 24 hours, and HO-1 expression was examined with Western blotting and qPCR. Results are expressed as the mean ± SEM. *P < 0.05 as compared with basal level. #*P *< 0.05 as compared with thrombin-treated group.

It has been reported that clotting factors and fibrinolytic products are increased in synovial fluid of patients with RA and OA [25]. Therefore, we examined the levels of thrombin expression in samples from patients with OA and found that the expression of thrombin protein in human OASFs (Figure 1A, lines 4 to 6) was significantly higher than that in normal SFs (Figure 1A, lines 1 to 3). The OASFs medium showed a level of expression of thrombin that was significantly higher than that seen in the medium from normal SFs (Figure 1B). In addition, concentrations of thrombin in synovial fluid were significantly higher in patients with OA than in controls (Figure 1B). We applied thrombin directly to OASFs to examine the expression of HO-1. Treatment of OASFs with thrombin (0.1 to 3 U/ml) for 24 hours induced HO-1 expression in a concentration-dependent manner (Figure 1C, E), and this induction occurred in a time-dependent manner (Figure 1D, F). After thrombin (3 U/ml) treatment for 24 hours, the amount of HO-1 expression had increased in OASFs (Figure 1D, F). To confirm further this stimulation-specific mediation by thrombin, PPACK, a thrombin inhibitor, was used. Pretreatment of cells with PPACK effectively antagonized the potentiating effect of thrombin on HO-1 expression (Figure 2A). These data indicated that thrombin increases HO-1 expression in human OASFs.

### Involvement of PAR1/PAR3 receptor in thrombin-mediated increase of HO-1 expression

Thrombin exerts its effects through interaction with specific PAR1, PAR 3, and PAR4 receptors [26]. To investigate the role of PAR1, PAR3, and PAR4 subtype receptors in thrombin-mediated increase of HO-1 expression, cells were treated with PAR1-, 3-, and 4-specific agonist peptides and then examined for expression levels of HO-1. Of the agonist peptides tested, the PAR1-selective receptor agonist peptide, SFLLRN-NH_2 _(100 μ*M*) and TFRGAP-NH_2 _(PAR3 agonist peptide; 100 μ*M*) significantly increased the expression of HO-1 (Figure 2A). In contrast, GYPGQV-NH_2 _(PAR4 agonist peptide; 100 μ*M*) failed to upregulate HO-1 expression. Furthermore, thrombin, SFLLRN-NH_2_, and TFRGAP-NH_2 _only slightly increased HO-1 expression in normal synovial fibroblasts (Figure 2B), indicating that PAR agonist-induced HO-1 expression is more important during OA pathogenesis. To confirm that PAR1 and PAR3 subtype receptors are involved in the thrombin-mediated increase of HO-1 expression, specific inhibition of PAR-receptor expression was accomplished with siRNA (Figure 2C). It was found that PAR1 and PAR3 receptor-specific siRNA but not PAR4-receptor siRNA significantly blocked the thrombin-mediated increase of HO-1 expression (Figure 2D, E), indicating that interactions between thrombin and PAR1/PAR3 are important for HO-1 expression in human OASF cells.

### The signaling pathways of PKCδ and c-Src are involved in the potentiating action of thrombin

**Figure 3:**
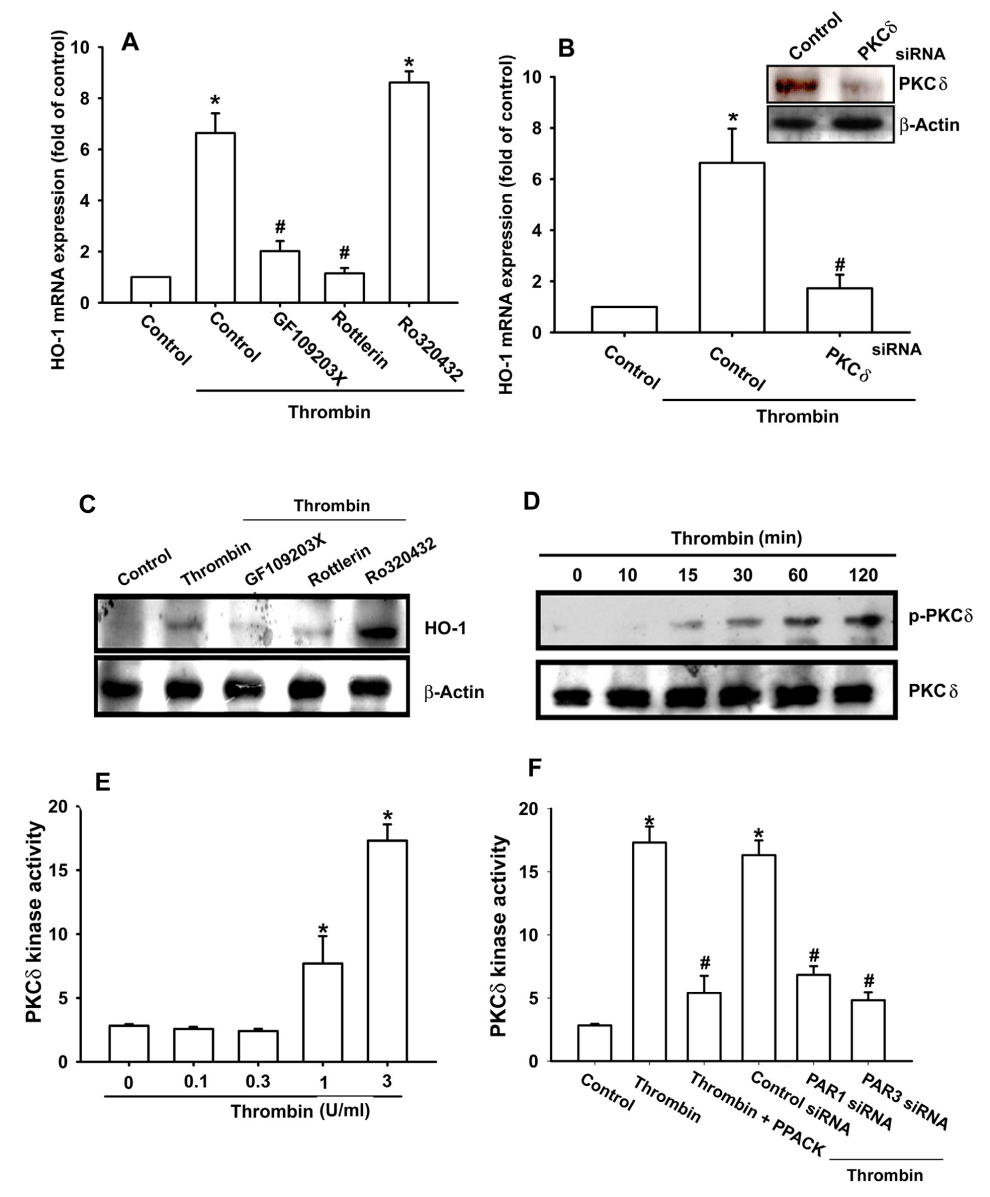
**PKCδ is involved in thrombin-induced HO-1 expression in synovial fibroblasts**. **(A, C) **Osteoarthritis synovial fibroblasts (OASFs) (six OA lines) were pretreated for 30 minutes with GF109203X (3 μ*M*), rottlerin (10 μ*M*), and Ro320432 (10 μ*M*) followed by stimulation with thrombin for 24 hours, and HO-1 expression was examined with Western blotting and qPCR. **(B) **Cells (seven OA lines) were transfected for 24 hours with PKCδ siRNA followed by stimulation with thrombin for 24 hours, and HO-1 expression was examined with qPCR. **(C) **Cells (six OA lines) were incubated with thrombin for indicated time intervals, and PKCδ phosphorylation was examined with Western blotting. **(D, E) **Cells (seven OA lines) were incubated with thrombin for 30 minutes or pretreated for 30 minutes with PPACK or transfected 24 hours with PAR1 and PAR3 siRNA, followed by stimulation with thrombin for 30 minutes, and PKCδ kinase activity was determined with the PKCδ kinase kit. Results are expressed as the mean ± SEM. *P < 0.05 as compared with basal level. #*P *< 0.05 as compared with thrombin-treated group.

PKC has been shown to play an important role in cellular functions modulated by several stimuli, including thrombin [27,28]. To determine whether PKC isoforms were involved in thrombin-triggered HO-1 expression, OASFs were pretreated for 30 minutes with either GF109203X, a pan-PKC inhibitor, or rottlerin, a selective PKCδ inhibitor [29], and then incubated with thrombin for 24 hours. As shown in Figure 3A and 3C, pretreatment with GF109203X and rottlerin but not PKCα inhibitor (Ro320432, 10 μ*M*) reduced thrombin-induced HO-1 expression, which suggests that PKCδ plays a specific role in thrombin-induced HO-1 expression in OASFs. Transfection of cells with PKCδ siRNA also reduced thrombin-induced HO-1 expression (Figure 3B). We directly measured the PKCδ phosphorylation response to thrombin. Stimulation of OASFs with thrombin led to a significant increase in phosphorylation of PKCδ (Figure 3D). The PKCδ activity in OASFs was increased by thrombin treatment in a dose-dependent manner (Figure 3E). Pretreatment of cells with PPACK or transfection of cells with PAR1 and PAR3 siRNA also reduced thrombin-mediated PKCδ kinase activity (Figure 3F). Based on these results, thrombin appears to act through a PAR1/PAR3 and PKCδ-dependent signaling pathway to enhance HO-1 expression in human synovial fibroblasts.

**Figure 4:**
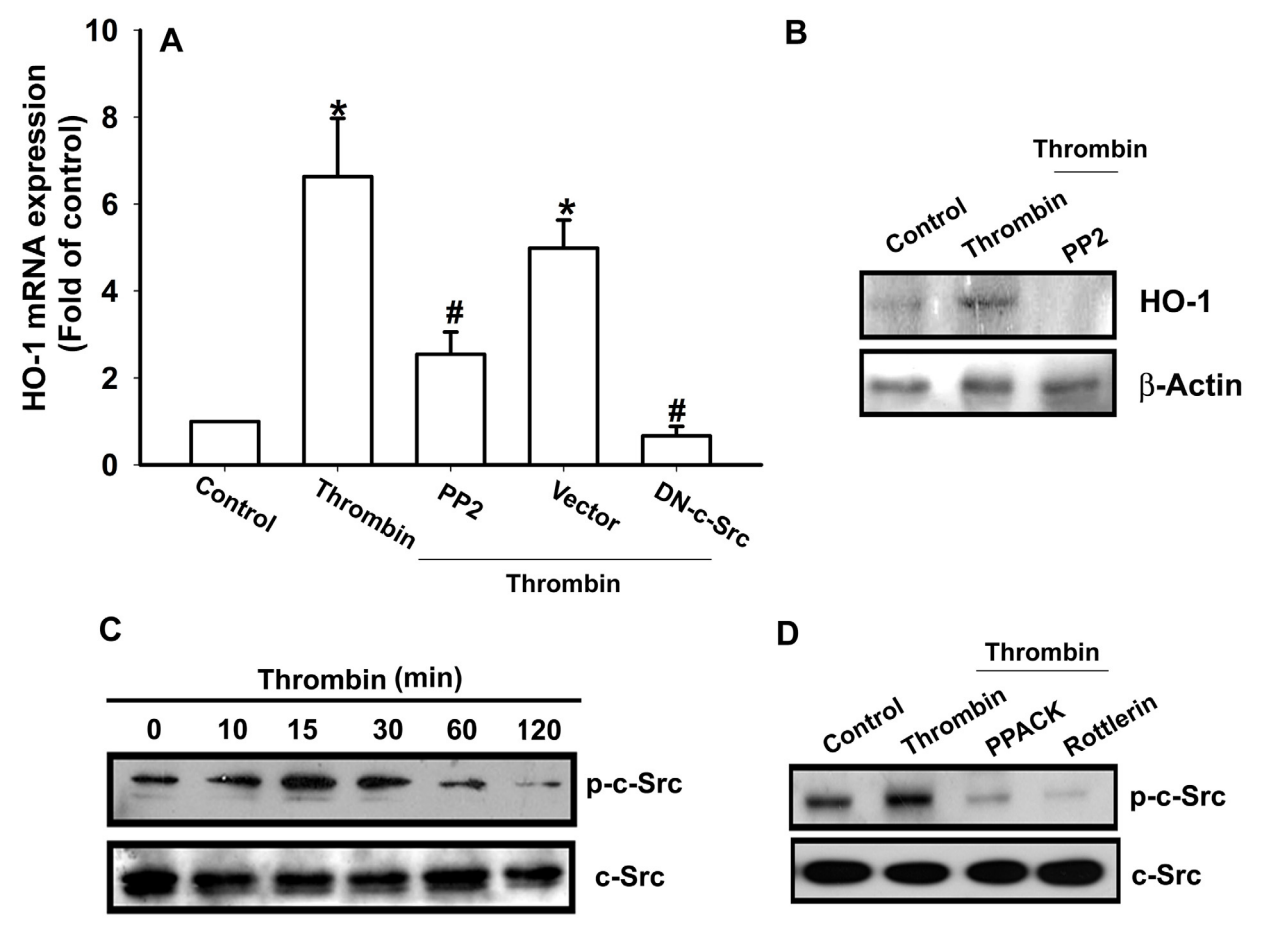
**c-Src is involved in thrombin-induced HO-1 expression in synovial fibroblasts**. **(A, B) **Osteoarthritis synovial fibroblasts (OASFs) (six OA lines) were pretreated for 30 minutes with PP2 (3 μ*M*) or transfected with c-Src mutant for 24 hours followed by stimulation with thrombin for 24 hours, and the HO-1 expression was examined with qPCR and Western blotting. **(C) **OASFs (five OA lines) were incubated with thrombin for indicated time intervals, and c-Src phosphorylation was examined with Western blotting. **(D) **Cells (six OA lines) were pretreated 30 minutes with PPACK or rottlerin, followed by stimulation with thrombin for 30 minutes, and c-Src phosphorylation was examined with Western blotting. Results are expressed as the mean ± SEM. *P < 0.05 as compared with basal level. #*P *< 0.05 as compared with thrombin-treated group.

PKCδ-dependent c-Src activation is involved in the regulation of COX-2 expression [30], and we investigated the role of Src in mediating thrombin-induced HO-1 expression with the specific Src inhibitor PP2. As shown in Figure 4A and 4B, thrombin-induced HO-1 expression was markedly attenuated by pretreatment of cells for 30 minutes with PP2 or by transfection of cells for 24 hours with c-Src mutant. The major phosphorylation site of c-Src at the Tyr^416 ^residue results in activation of c-Src autophosphorylation [31]. To examine directly the crucial role of c-Src in HO-1 expression, we measured the level of c-Src phosphorylation at Tyr^416 ^in response to thrombin. As shown in Figure 4C, treatment of OASFs with thrombin resulted in a time-dependent phosphorylation of c-Src at Tyr^416^. Pretreatment of cells with PPACK and rottlerin markedly inhibited the thrombin-induced c-Src kinase activity (Figure 4D). Based on these results, thrombin appears to act through a signaling pathway involving the PAR1/PAR3 receptor, PKCδ, and c-Src to enhance HO-1 expression in human synovial fibroblasts.

### Involvement of Nrf2 in thrombin-induced HO-1 expression

**Figure 5:**
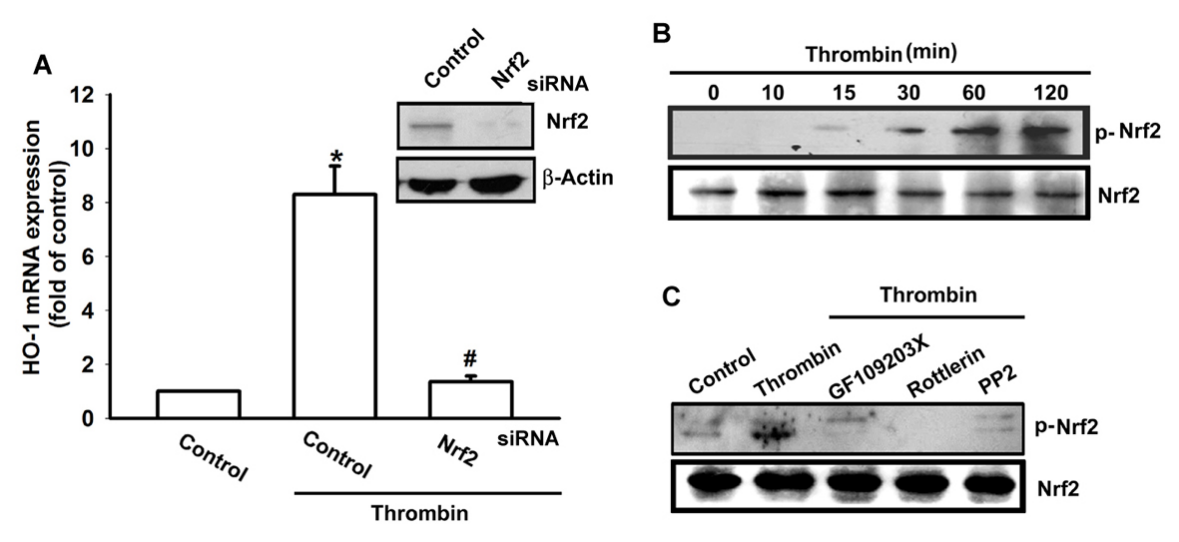
**Thrombin induced Nrf2 activation in synovial fibroblasts**. **(A) **Osteoarthritis synovial fibroblasts (OASFs; six OA lines) were transfected with Nrf2 siRNA for 24 hours, and HO-1 expression was examined with qPCR. **(B) **OASFs (six OA lines) were incubated with thrombin for indicated time intervals, and Nrf2 phosphorylation was examined with Western blotting. **(C) **Cells (five OA lines) were pretreated 30 minutes with PPACK, rottlerin, or PP2 followed by stimulation with thrombin for 30 minutes, and Nrf2 phosphorylation was examined with Western blotting. Results are expressed as the mean ± SEM. *P < 0.05 as compared with basal level. #*P *< 0.05 as compared with thrombin-treated group.

Activation of Nrf2 has been reported to play an important role in HO-1 expression [32]. To determine the role of Nrf2 in thrombin-mediated HO-1 expression, OASFs were transfected with Nrf2 siRNA. Transfection of cells with Nrf2 siRNA suppressed the thrombin-induced HO-1 expression (Figure 5A). Stimulation of cells with thrombin also induced Nrf2 phosphorylation in a time-dependent manner (Figure 5B). Pretreatment of cells with GF109203X, rottlerin, and PP2 attenuated thrombin-induced Nrf2 activation (Figure 5C).

**Figure 6:**
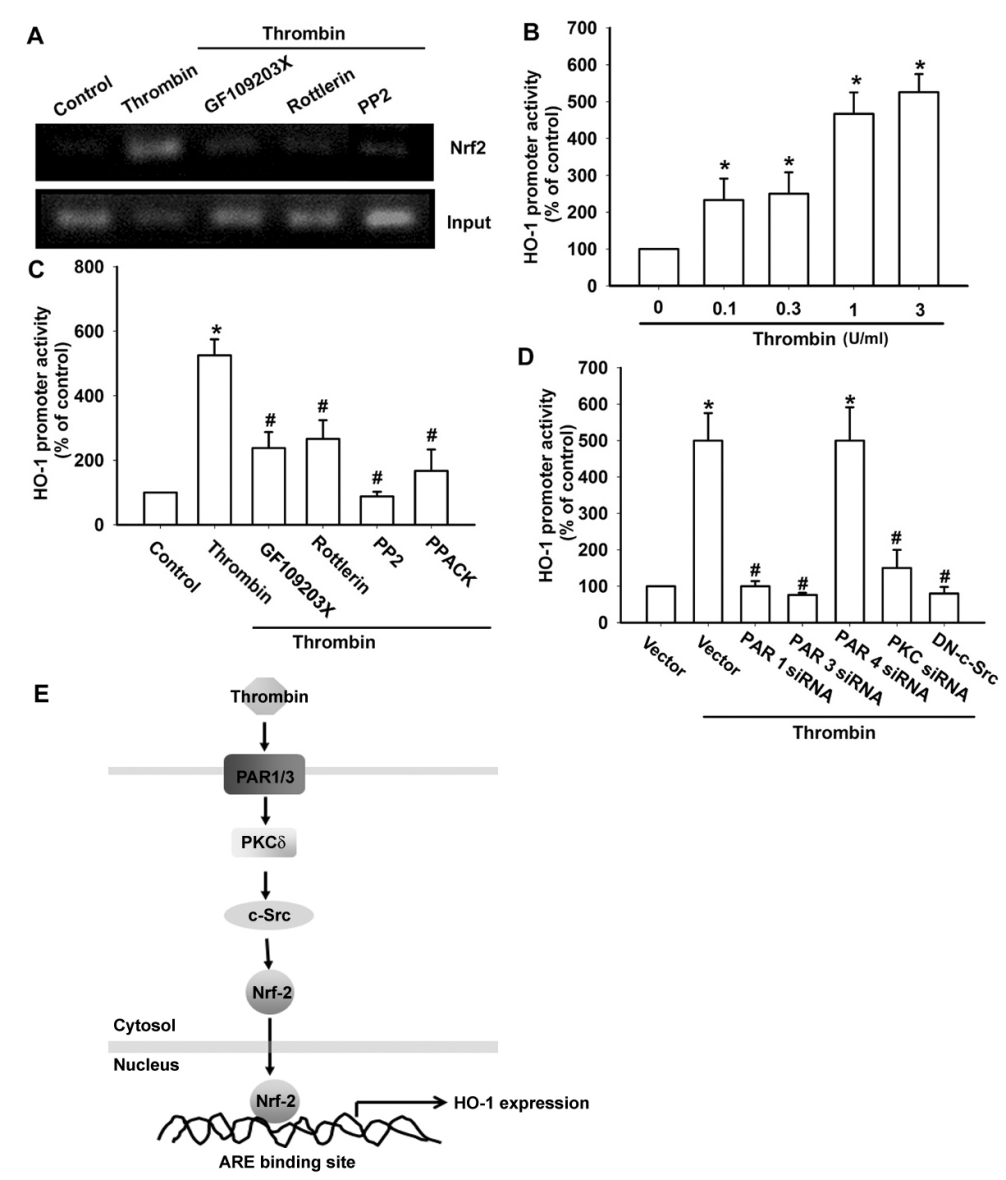
**PAR, PKCδ, and c-Src are involved in thrombin-induced Nrf2 activation**. **(A) **Osteoarthritis synovial fibroblast (OASFs; seven OA lines) were pretreated with GF109203X, rottlerin, and PP2 for 30 minutes followed by stimulation with thrombin for 120 minutes, and ChIP assay was then performed. Chromatin was immunoprecipitated with anti-Nrf2 antibody. One percent of the precipitated chromatin was assayed to verify equal loading (Input). OASFs (eight OA lines) were incubated with thrombin for 24 hours **(B) **or pretreated with PPACK, GF109203X, rottlerin, and PP2 for 30 minutes **(C) **or co-transfected with PAR1 siRNA, PAR3 siRNA, PAR4 siRNA, PKCδ siRNA, and c-Src mutant for 24 hours **(D) **before incubation with thrombin for 24 hours. HO-1 luciferase activity was then assayed. Results are expressed as the mean ± SEM. *P < 0.05 as compared with basal level. #*P *< 0.05 as compared with thrombin-treated group. **(E) **Schematic diagram of the signaling pathways involved in thrombin-induced HO-1 expression in OASFs. Thrombin increases HO-1 expression by binding to the PAR1/PAR3 receptor and activating PKCδ and c-Src, which enhances binding of Nrf2 to the ARE site. This results in the transactivation of HO-1 expression.

We next investigated whether Nrf2 binds to the ARE element on the HO-1 promoter after thrombin stimulation. The *in vivo *recruitment of Nrf2 to the HO-1 promoter (-857 to -752) was assessed with chromatin immunoprecipitation assays. *In vivo *binding of Nrf2 to the ARE element of the HO-1 promoter occurred after thrombin stimulation (Figure 6A). The binding of Nrf2 to the ARE element by thrombin was attenuated by GF109203X, rottlerin, and PP2 (Figure 6A).

To study further the pathways involved in the action of thrombin-induced HO-1 expression, transient transfection was performed by using the human HO-1 promoter-luciferase construct. Treatment of cells with thrombin led to dose-dependent increases in HO-1 promoter activity (Figure 6B). PPACK, GF109203X, rottlerin, and PP2; PAR1, PAR3, and PKCδ siRNA; and c-Src mutant all antagonized the stimulation of HO-1 promoter activity by thrombin (Figure 6C, D). These results indicated that thrombin-induced HO-1 expression is mediated through the PAR1/PAR3, PKCδ, c-Src, and Nrf2 pathways in OASFs.

## Discussion

OA is a heterogeneous group of conditions associated with defective integrity of articular cartilage and related changes in the underlying bone. The chronic inflammatory process is mediated through a complex cytokine network. It is not yet completely understood which factors are responsible for initiating the degradation and loss of articular tissues. It has been shown that thrombin acts as a mitogen to stimulate the abnormal proliferation of synovial cells during RA and OA pathogenesis [14,15]. We hypothesized that thrombin is highly expressed during OA pathogenesis and promotes the release of inflammatory cytokines as well as increased HO-1 production (an endogenous compensation mechanism). First, we confirmed that synovial fluid concentrations of thrombin were significantly higher in patients with OA than in normal fluid samples. We then identified HO-1 as a target protein for the thrombin-signaling pathway in OASF cells. Our results provided evidence that thrombin promotes the endogenous compensation mechanism (that is, HO-1 expression), during OA pathogenesis. We found that thrombin increased HO-1 production (the antiinflammatory response); however, this mechanism is insufficient to arrest the progress of OA. Nonetheless, the discovery of the HO-1 signaling pathway may help us to develop effective therapy in the future.

Thrombin is known to activate three PARs, including PAR1, PAR3, and PAR4 [33]. However, we demonstrated that PAR1 and PAR3 but not PAR4 receptors were required for thrombin-induced HO-1 production. Treatment with PAR1 or PAR3 agonist induced HO-1 expression, but PAR4 agonist failed to upregulate HO-1 expression. Furthermore, we could not inhibit thrombin-induced HO-1 upregulation by PAR4 receptor-specific siRNA. These data suggest that PAR1 and PAR3 receptors are involved in thrombin-induced HO-1 expression in human synovial fibroblasts.

Several isoforms of PKC have been characterized at the molecular level and have been found to mediate a variety of cellular molecular responses [34]. We demonstrated that the PKC inhibitor GF109203X antagonizes the thrombin-mediated potentiation of HO-1 expression, suggesting that PKC activation is an obligatory event in thrombin-induced HO-1 expression in these cells. In addition, rottlerin but not Ro320432 also inhibited thrombin-induced HO-1 expression.

One current report indicates that rottlerin is not a specific PKCδ inhibitor but inhibits many other targets [35]. Therefore, we used PKCδ siRNA to confirm PKCδ function in OASFs. We found that PKCδ siRNA inhibited the enhancement of HO-1 expression. Incubation of synovial fibroblasts with thrombin also increased PKCδ phosphorylation and kinase activity. Conversely, PPACK and PAR1 or PAR3 siRNA reduced thrombin-mediated PKC kinase activity. These data suggest that the PAR1/PAR3 and PKCδ pathways are required for thrombin-induced HO-1 expression.

Src, a tyrosine kinase, plays a critical role in the induction of chemokine transcription [36]. Because c-Src is a downstream effector of PKCδ [30], we examined the potential role of c-Src in the signaling pathway of thrombin-induced HO-1 expression. Treatment of cells with c-Src inhibitor PP2 or transfection of cells with c-Src mutant reduced thrombin-mediated HO-1 expression. In addition, we found that treatment of OASFs with thrombin promoted increases in c-Src phosphorylation. These effects were inhibited by PPACK and rottlerin, indicating the involvement of PKCδ-dependent c-Src activation in thrombin-mediated HO-1 induction.

Several binding sites exist for a number of transcription factors, including ARE in the 5' promoter region of the *HO-1 *gene [37]. The results of this study show that Nrf2 activation contributes to thrombin-induced HO-1 production in synovial fibroblasts, and that inhibition of the Nrf2-dependent signaling pathway, including Nrf2 siRNA, inhibits thrombin-induced HO-1 expression. We found that treatment of synovial fibroblasts with thrombin resulted in increased Nrf2 phosphorylation. Furthermore, thrombin increased the binding of Nrf2 to the ARE element within the HO-1 promoter, as shown by a chromatin immunoprecipitation assay. Binding of Nrf2 to the ARE element was attenuated by GF109203X, rottlerin, and PP2. By using transient transfection with HO-1-luciferase as an indicator of HO-1 activity, we also found that thrombin induced an increase in HO-1 activity. In addition, PPACK, GF109203X, rottlerin, and PP2 reduced thrombin-increased HO-1 promoter activity. Based on these findings, we propose that the PAR1/PAR3, PKCδ, and c-Src pathways are involved in thrombin-induced Nrf2 activation in human synovial fibroblasts.

## Conclusions

We explored the signaling pathways involved in thrombin-induced HO-1 expression in human synovial fibroblasts. We found that thrombin augmented HO-1 expression by binding to the PAR1/PAR3 receptor and activating PKCδ and c-Src, which enhanced binding of Nrf2 to the ARE site and resulted in HO-1 expression (Figure 6E). The discovery of this HO-1 signaling pathway helps us to understand the mechanism of OA pathogenesis and may lead us to develop effective therapy in the future.

## Abbreviations

ChIP: chromatin immunoprecipitation; ELISA: enzyme-linked immunosorbent assay; HO-1: heme oxygenase-1; IL: interleukin; MMP: matrix metalloproteinase; OA: osteoarthritis; OASF: osteoarthritis synovial fibroblast; PAR: protease-activated receptor; qPCR: quantitative real-time polymerase chain reaction; RA: rheumatoid arthritis; TNF: tumor necrosis factor.

## Competing interests

The authors declare that they have no competing interests.

## Authors' contributions

JFL performed the experiments. SMH, CYH, CHT, and WHY analyzed the data and provided the suggestions. CTT conceived of and designed the experiments. All authors read and approved the final manuscript.
